# A perspective on the molecular mechanism in the control of organ internal (IN) asymmetry during petal development

**DOI:** 10.1093/hr/uhac202

**Published:** 2022-09-06

**Authors:** Qianxia Yu, Liangfa Ge, Sagheer Ahmad, Da Luo, Xin Li

**Affiliations:** Department of Grassland Science, College of Forestry and Landscape Architecture, South China Agricultural University, Guangzhou 510642, China; Department of Grassland Science, College of Forestry and Landscape Architecture, South China Agricultural University, Guangzhou 510642, China; Guangdong Academy of Agricultural Sciences, Guangzhou 510642, China; Key Laboratory of Soybean Molecular Design Breeding, Northeast Institute of Geography and Agroecology, Chinese Academy of Sciences, Harbin 150081, China; College of Life Sciences, Nanjing Agricultural University, Nanjing 210095, China

## Abstract

Floral zygomorphy (monosymmetry) is a key innovation in flowering plants and is related to the coevolution of plants and their animal pollinators. The molecular basis underlying floral zygomorphy has been analysed, and two regulatory pathways have been identified: one determines the dorsoventral (DV) asymmetry along the floral plan, and the other controls organ internal (IN) asymmetry during petal development. While strides have been made to understand the molecular mechanism controlling DV asymmetry, which mainly involves an interplay between TCP and MYB transcription factors, the molecular pathway regulating IN asymmetry remains largely unknown. In this review, we discuss what is known about regulators and the molecular pathway regulating IN asymmetry. Our analysis revealed that the regulation of IN asymmetry occurs at the cellular, tissue, and organ genesis levels during petal development and that the regulatory mechanism is likely integrated into different developmental paths, such as floral and root nodule development. Although the molecular regulation of IN asymmetry is not be a linear path, a key hub for the regulatory network could be vascular patterning during petal organogenesis.

## Introduction

Symmetry has always fascinated humans in art and natural science, such as mathematics and biology [1]. An important symmetric form in biology is the construction of live organisms. During the course of evolution, symmetry is involved in the developmental systems for all levels of biological organization and integration, including molecules, cells, tissues, organs, and whole organisms.

In plants, the most popular or attractive discussions regarding symmetry pertains to the flower. Floral symmetry plays an important role in the preference for and perception of flowers by pollinators; therefore, it is considered to be strongly correlated with plant–pollinator systems [2–4]. Studies on floral symmetry have mainly focused on the developmental and genetic bases of the overall symmetry of flower. However, the mechanisms regulating symmetry in certain floral organs (e.g. petal, style, and petaloid staminode) have been largely ignored.

**Figure 1:**
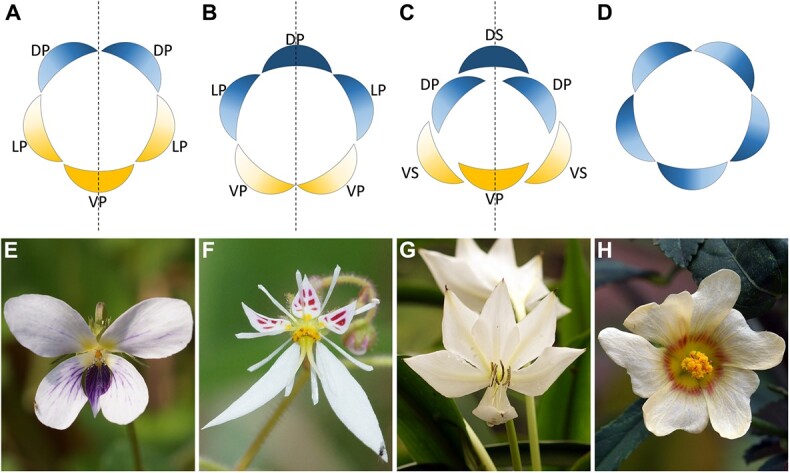
Different patterns of internal asymmetry in petals. **A** and **B** Pentamerous zygomorphic flowers. **C** Trimerous zygomorphic flower with two whorls of tepals; in some cases, they are differentiated into an outer whorl of three sepals and an inner whorl of three petals. **D** Pseudo-actinomorphic flower with all petals internally asymmetrical caused by contort aestivation. **E**–**H** Examples for floral patterns in (**A**–**D**), respectively. **E***Viola stewardiana* (Violaceae). **F***Saxifraga stolonifera* (Saxifragaceae). **G***Crinum moorei* (Amaryllidaceae). **H***Sida rhombifolia* (Malvaceae). DP, dorsal petal; DS, dorsal sepal; LP, lateral petal; VP, ventral petal; VS, ventral sepal. Dotted lines in (**A**–**C**) indicate the symmetry planes of zygomorphic flowers. Petals with gradient color in (**A**–**D**) indicate internal asymmetry.

Organ internal (IN) asymmetry in plants, which refers to the left and right halves of an organ displaying distinct shapes, is thought to have evolved multiple times independently (Fig. 1). The asymmetrical organs usually occur in two mirror-image forms: left-handed and right-handed. In many cases, these two mirror-image forms may either occur in the same flower in opposite directions along the pedicel (e.g. the paired lateral petals and dorsal petals in *Antirrhinum*) or sometimes appear in different flowers on the same plant (e.g. the dorsal stamen with half fertile anther and half petaloid staminodes in paired flowers of *Thalia*) [5]. In some cases, the two asymmetrical forms are found in different individuals (e.g. the style in *Heteranthera multiflora*) [3]. This suggests the potential co-relation between organ internal asymmetry and handedness in plants.

Organ internal asymmetry is supposed to occur randomly during the course of evolution. However, not all of its characteristics can be inherited in modern plants after natural selection. Those that have been inherited over generations are usually biologically functional. Interestingly, organ internal asymmetry can contribute to the overall asymmetry of a flower, which may interact with bees to influence the breeding system. Moreover, an asymmetrical organ itself may also play a significant role in plant pollination. For example, the left and right styles in *H. multiflora* are thought to promote cross-pollination [3].

It appears that plants can precisely produce the left- or right-handed organs in the right position. Thus, the regulators affecting IN should have been connected to a stable system that plays a basic role in controlling the asymmetrical growth of cells and organs under different conditions.

## The forms and distribution of petal internal asymmetry across angiosperms

Internal asymmetry in petals is prevalent among different clades of flowering plants. The most common examples of IN occur in zygomorphic flowers. Contributing to the strong bilateral symmetry of the flower, the paired petals located opposite the pedicel are internally asymmetrical due to different growth patterns. Four types of petal internal asymmetry are listed according to the patterns of floral symmetry:

(i) In pentamerous zygomorphic flowers, such as Plantaginaceae, Acanthaceae, Lamiaceae, Gesneriaceae, Balsaminaceae, and Violaceae, paired dorsal and lateral petals are internally asymmetrical and mirror images of each other, while the single ventral petal is symmetrical (Fig. 1A and E).
(ii) In another pattern of pentamerous zygomorphic flowers, one dorsal petal is bilaterally symmetrical, and the two lateral petals and two ventral petals are individually asymmetrical and mirror images of each other (Fig. 1B and F). Examples can be found in Fabaceae, Saxifragaceae, and Ericaceae.
(iii) Trimerous zygomorphic flowers are usually found in monocots, wherein six tepals (sometimes differentiated into three sepals and three petals) are arranged in two whorls. The dorsal sepal (the dorsal tepal in the outer whorl) and ventral petal (the ventral tepal in the inner whorl) are bilaterally symmetrical, and the remaining four members are internally asymmetrical (Fig. 1C and G). These phenomena are obvious in some clades, such as Orchidaceae and Amaryllidaceae.
(iv) In many cases, asymmetrical petals are also correlated with contort aestivation (Fig. 1D and H). The petal internal asymmetry is especially conspicuous in some species of Apocynaceae (*Vinca*, *Cerbera*, *Kopsia*, *Tabernaemontana*, and *Trachelospermum*), Malvaceae (*Sida*, *Malva*, *Hibiscus*, *Kielmeyera*, and *Dombeya*), Calophyllaceae (*Kielmeyera*), Linaceae (*Linum*), Oxalidaceae (*Oxalis*), Plumbaginaceae (*Plumbago*), and Rubiaceae (*Gardenia*) [6]. In these lineages, flowers have a spiral phyllotaxis. Flower petals are contorted in the same direction as the phyllotaxis of the whole plant, making the individual petals asymmetric. However, the petals are arranged symmetrically; thus, the flower as a whole is actinomorphic. This kind of floral symmetry has sometimes been called pseudo-actinomorphy [6].

## Factors determining IN asymmetry

**Figure 2:**
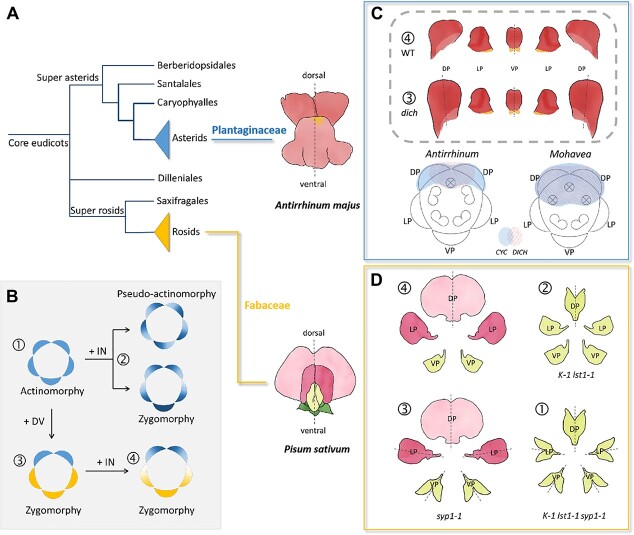
Factors determining organ internal (IN) asymmetry. **A** The simplified phylogenetic tree of core eudicots showing the relationship between the two species used for IN studies: *Antirrhinum majus* and *Pisum sativum*. **B** Diagrams showing the transits between different flower forms, adopted and modified from [14]. The petals with a gradient color indicate internal asymmetry. Petals of pure color are bilaterally symmetrical. **C** The role of *DICH* in dorsal petal morphology in *Antirrhinum* and *Mohavea* flowers. The dorsal petal in the *dich* mutant is more symmetrical than that of the wild-type plant in *Antirrhinum* [8]. The expressions of *CYC* and *DICH* are broader in *Mohavea* than in *Antirrhinum,* which is consistent with the morphological differences in petals and stamens between these two species. **D** The interplay of *SYP1* and TCP factors (*K* and *LST*) controlling DV and IN asymmetry in the *P. sativum* flower. In the *k-1 lst-1* mutant, all petals resemble the ventral one, but the flower is still zygomorphic because the IN asymmetry in the petal is not eliminated. In the *syp1–1* mutant, all petals are more symmetrical than those of wild type, and the flower still has the dorsoventral identity. In the *k lst1 syp1–1* triple mutant, the flower is actinomorphic, with all petals resembling the ventral one, and they are totally bilateral symmetric [14]. The numbers in circles represent the floral patterns summarized in (**B**).

Research on molecular mechanisms controlling petal internal asymmetry during petal development has primarily been carried out in Plantaginaceae and Fabaceae, two families with marked pentamerous zygomorphic flowers. Plantaginaceae and Fabaceae belong to two main lineages in core eudicots: Asterids and Rosids, respectively. Petals in these two families are arranged in two different patterns (Fig. 1A–B, 2A).

**Table 1:** The factors controlling petal internal asymmetry in previous studies.

**IN** **factors**	**Gene family**	**Ortholog/closest gene in *Arabidopsis***	**Species studied**	**Mutant phenotype**	**Other Functions**	**Reference**
*DICH*	TCP	*TCP1*	*Antirrhinum majus*; *Mohavea confertiflora*	The dorsal petals of *dich* are more symmetrical than that of WT in *A. majus*.	Stamen development; floral symmetry.	[8, 12]
*SYP1*	ALOG	*LSH3*	*Pisum sativum*	Symmetrical lateral and ventral petals.	Inflorescence development; spikelet morphogenesis, grain shape, and yield in rice; organ boundary formation; flower and leaf development; nodulation.	[14]
*BIO*	KIX domain	*KIX8*/*KIX9*	*P. sativum*	Symmetrical lateral and ventral petals; enlarged leaves and petals.	Regulation of organ size.	[18]
*ELE1*	TIFY	*PPD1*/*PPD2*	*P. sativum*	Symmetrical lateral and ventral petals; enlarged leaves and petals.	Regulation of organ size.	[17, 18]
*COCH*	NBCL	*BOP*	*P. sativum*	Symmetrical lateral and ventral petals.	Regulation of compound leaf and nodule identity in pea.	[19, 25, 26]
*LOW*	Dof	*AtDof3.4*/*AtDof5.8*	*Vigna radiata*	Changes in the symmetry of lateral and ventral petals, which therefore result in the deformed landing platform formation.	Regulation of development and function of vascular tissues.	[27]

### *DICH* is responsible for the internal asymmetry of dorsal petal in *Antirrhinum majus* and its closely related species *Mohavea confertiflora*

*A. majus*, bearing typical zygomorphic flowers, is used as a model system for flower symmetry. In wild type *A. majus*, petals along the dorsoventral axis have different shapes: one ventral petal is bilaterally symmetrical, while two dorsal petals and two lateral petals are individually asymmetrical (Fig. 2B). Research has demonstrated that the dorsoventral (DV) asymmetry of *Antirrhinum* flower is determined by a combination of some key regulators. CYCLOIDEA (CYC), DICHOTOMA (DICH), and RADIALIS (RAD) primarily control dorsal organ identity [7–9]. Two MYB proteins, DIVARICATA (DIV) and DIV-and-RAD-Interacting-Factor (DRIF), are required for ventral organ identity [9, 10].

CYC, a member of the TCP (TB1, CYC, and PCFs) family of transcription factors, was the first floral symmetry regulator identified in plants [7]. *CYC* is expressed in the dorsal region of flowers and plays a role in dorsal stamen abortion and dorsal petal identity. Mutation in *cyc* alone produces flowers with a semi-peloric phenotype in which the lateral petals are ventralized, while the dorsal petals exhibit combined characteristics of dorsal and lateral petals. This means that *CYC* alone cannot determine the dorsal identity of the flower, and other factors are involved in this process. *DICH* has been identified as a factor controlling internal asymmetry of dorsal petals [8]. The *dich* mutant exhibits a more subtle phenotype, with more symmetrical dorsal petal lobes, compared to that of the wild-type plants. The *cyc dich* double mutant has radially symmetrical flowers, with all the petals fully ventralized. Thus, both *CYC* and *DICH* are needed to determine dorsal identity. As a close homolog to *CYC*, *DICH* has an expression pattern similar to that of *CYC* at the early floral developmental stages, which is expressed in the dorsal region of the floral primordium. However, in the late developmental stages, the expression of *DICH* is restricted to the dorsal parts of the dorsal petals [8]. Taken together, the asymmetrical expression of *DICH* across dorsal petals results in the internal asymmetry of dorsal petals [8], perhaps through differential regulation of cell division and expansion [11].

The role of DICH in petal internal asymmetry has been verified in another Plantaginaceae species, *M. confertiflora*, a close relative of *A. majus*. The flower of *M. confertiflora* differs from that of *A. majus* in its dorsal petal shape and stamen number. It has been shown that a higher degree of dorsal petal internal symmetry in *Mohavea* is correlated with the expanded expression of *DICH* homologs, resulting in the uniform expression of *DICH* homologs across dorsal petals (Fig. 2B) [12]. Therefore, it may change the downstream genes that function in cell division and expansion during petal differentiation, resulting in the alternation of the petal shape [13].

### SYP1, the main factor determining petal IN asymmetry in Fabaceae

In legumes (Fabaceae), petal internal asymmetry is prominent in the subfamily Papilionoideae bearing papilionaceous corolla (butterfly corolla). *Pisum sativum* produces typical papilionaceous flowers. The five petals are arranged along the dorsoventral axis with one symmetrical dorsal petal, two lateral petals, and two ventral petals, which are internally asymmetrical (Fig. 2D).

In Fabaceae, the IN asymmetry of the lateral and ventral petals is not regulated by TCP factors. When mutating or silencing the expression of *CYC* homologs (*LOBED STANDARD* and *KEELED WINGS*), all petals in the flower resemble the ventral petal in the wild type but still display intact IN asymmetry (Fig. 2D) [14–16].

It has been shown that the key factor determining petal organ internal asymmetry is the *SYMMETRIC PETALS 1* (*SYP1*) gene, which is a member of the ALOG (*Arabidopsis* LSH1 and *Oryza* G1) family of proteins. In the *syp1* mutant (*syp1–1*), almost all petals are symmetrical. The symmetric phenotype is especially conspicuous in ventral petals. Each ventral petal in the *syp1–1* mutant can form a keel-like structure, while in the wild-type plant, the keel is formed by two ventral petals (Fig. 2D). When *syp1–1* is introduced into *k-1 lst1–1*, the flowers of triple mutants display radial symmetry, and all petals possess a ventralized identity with a keel-like structure (Fig. 2D). Thus, a default form without DV and IN asymmetries is identified in peas, suggesting that DV and IN asymmetry in legume flowers is independently regulated by different factors.

**Figure 3:**
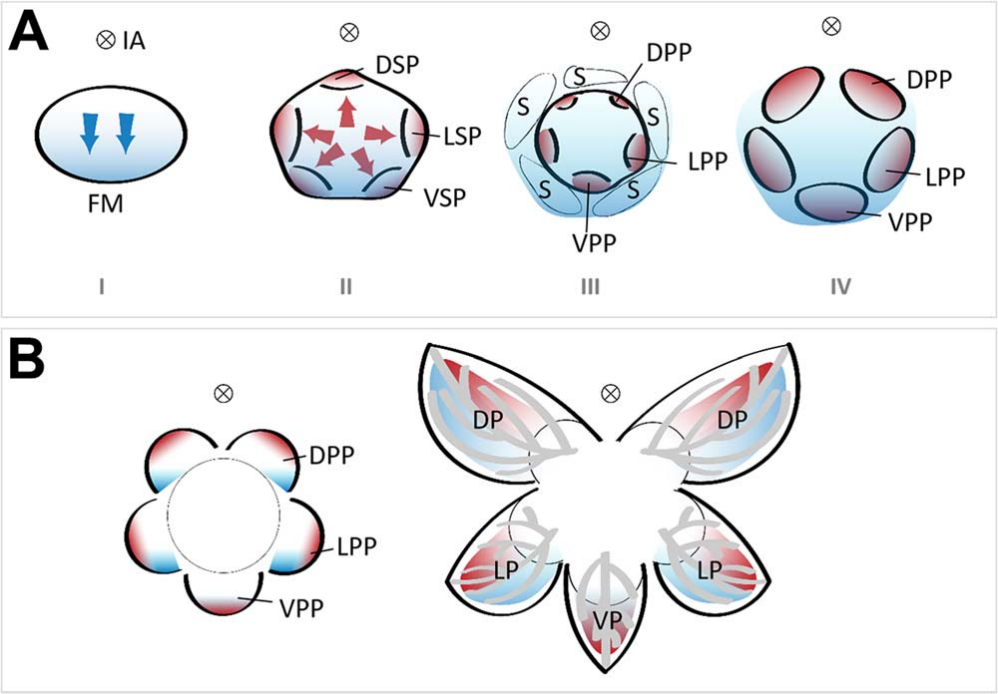
The hypothesized spatial and temporal auxin distribution contributes to asymmetric petal formation in zygomorphic flowers. **A** Diagrams show the early floral development stages. Stage I: blue color indicates the dorsoventral auxin concentration gradient established when floral meristem is initiated from the periphery of the inflorescence meristem. Stage II: red color indicates the maximum auxin concentration zone where sepal primordia is initiated on the floral meristem. Stage III: petal primordia is initiated. Red colors indicate the maximum auxin concentration at the tips of the petal primordia. Stage IV: auxin gradient patterning in petal primordia. **B** Petal internal asymmetry as a developmental constraint imposed by auxin-dependent vascular patterning. DP, dorsal petal; DPP, dorsal petal primordium; DSP, dorsal sepal primordium; FM, floral meristem; IA; inflorescence axis; LP, lateral petal; LPP, lateral petal primordium; LSP, lateral sepal primordium; S, sepal; VP, ventral petal; VPP, ventral petal primordium; VSP, ventral sepal primordium. Blue and red arrows indicate the division directions to initiate new primordia. The gradient of the blue and red colors indicates the concentration of auxin: the dark color represents maximum auxin concentration while the light color represents minimum auxin concentration.

### Other factors involved in the organ internal asymmetry in legumes

Further studies in pea and another legume species, *Vigna radiata* (mungbean), recognized that, other than *SYP1*, there are multiple factors involved in organ internal asymmetry. These factors include *ELEPHANT EAR-LIKE LEAF1* (*ELE1*), *BIGGER ORGANS* (*BIO*), *COCHLEATA* (*COCH*), and *LOVE ON WINGS* (*LOW*) (Table 1). Mutations in these genes all produce flowers with defective IN asymmetry similar to that of the *syp1* mutant [17–19].

*ELE1* and *BIO* were identified from *ele1* and *bio* mutants. Other than the alternation in petal shape, the *ele1* and *bio* mutants also have enlarged organs [17, 18]. Research has shown that ELE1 is a member of the TIFY family proteins and is an ortholog of *Arabidopsis* PEAPOD1 (PPD1) and PPD2 [20–22]. BIO is orthologous to AtKIX8/9 protein, and it belongs to the KIX domain family proteins [23]. BIO could physically interact with ELE1, and the BIO-ELE1 module along with the LATHYROIDES (LATH) represses the expression of its downstream genes, such as *GROWTH-REGULATING-FACTOR 5*, thereby controlling the shape and size of the leaf and petal [18, 24]. In addition, it is supposed that the effects of *BIO* on petal asymmetry may be achieved through the same genetic pathway of IN asymmetry regulated by *SYP1* because the *syp1–1 bio-1* double mutants display effects on the IN asymmetry of the lateral and ventral petals similar to those of *syp1–1* and *bio-1* single mutants [18].

COCH is an ortholog of *Arabidopsis* BLADE-ON-PETIOLE (BOP), belonging to the NBCL gene family, and it is known as a key component in the control of compound leaf and nodule development in peas [25, 26]. COCH regulates floral organ internal asymmetry based on its physical interaction with SPY1. *COCH* has an expression pattern similar to that of *SYP1*, and the *coch* mutant shows an abnormal flower similar to that of the *syp1* mutant that has symmetrical lateral and ventral petals [19]. Biochemical analysis has revealed that COCH represses degradation of SYP1 mediated by the 26S proteasome and therefore promotes SYP1 protein stability [19].

Moreover, *love on wings* (*low*) is a mutant of *V. radiata,* screened by large-scale gamma-ray mutagenesis [27]*.* The *low* mutant shows deformed landing platform formation and has developmental defects in the vasculature. By dissecting the opened flower, the researchers have found changes in the shape of both the lateral and ventral petals. The lateral petals in the *low* mutant are more symmetrical than those of the wild-type flower. Moreover, similar to the phenotype of the *syp1* mutant in pea, in the flowers of the *low* mutant, a single ventral petal developed into a keel-like structure [27]. Genetic analysis has revealed that *LOW* encodes a Dof-like protein localized in the nucleus, which is expressed in the provasculature of flowers and other tissues [27]. This shows that the changes in petal asymmetry in the *low* mutant are linked to defects in the petal vasculature.

### The connections between the two parallel systems: snapdragon and legumes

Multiple factors in different developmental paths have been found to participate in petal internal asymmetrical growth in snapdragon and legume species. However, researchers have not found any key factors that are crucial in petal internal asymmetry regulation in these parallel systems. This indicates that the IN pathway may not be linear. Even so, we can still find some common traits between these two research systems. First, the flowers in these two systems are produced in a lateral position in an indeterminate inflorescence. The floral meristems are placed in a similar asymmetric developmental environment established by physical stress and plant hormone concentrations from a developmental perspective. This, to some extent, can explain why organ internally asymmetry is stabilized in bilateral flowers. In addition, researchers have mentioned that petal venation patterns are altered in *dich* and *low* mutants of snapdragon and mungbean compared with that of the wild-type asymmetrical petals [8, 27]. Thus, the vascular system should be key in the IN regulation path, which needs further clarification.

## Vascular patterning and the IN pathway in leaf-like organs

In angiosperms, the final shape of a leaf-like organ (e.g. leaf and petal) appears to be closely correlated with its vascular patterns [28, 29]. Current analyses of mutant phenotypes provide strong support that the pattern and ontogeny of leaf venation appear to guide or limit many aspects of leaf cell division and differentiation [30]. During the process of leaf development, multiple transcription factors, signal peptides, and phytohormones are involved in the complex networks that regulate the formation of vascular tissues [31–34]. A role for auxin in this process was identified many years ago, which is believed to be a universal basic mechanism governing the spatial regulation of vein pattern formation [34]. Mutation in the auxin signaling pathway disturbs vein patterning, resulting in an alternation of leaf shape [35–39].

As a homologous organ to the leaf, the morphogenesis of a flower petal is also coordinated with its vasculature [8, 27]. However, the conclusion is only drawn from limited models, and the nature of this relationship remains obscure in other plants. Relatively little is known about the role of auxin in the developmental regulation of petal morphogenesis, vascular pattern formation, and the relationship between them. By comparing the general IN pathway in leaf development, we can propose a model for the petal IN regulation pathway in zygomorphic flowers (Fig. 3). As already known, zygomorphic flowers are found in the lateral position of an inflorescence [40–42]. The new flower primordia emerges in the peripheral zone of the shoot apical meristem (SAM) with a maximum concentration of auxin [43]. The auxin gradient along the dorsoventral axis influences the expression of corresponding symmetry genes and guides the process of cell proliferation, leading to the initial zygomorphic floral ground plan (Fig. 3A; Stage I, marked with blue gradient color). The auxin distribution pattern changes with the initiation of the first whorl of the floral organs. The maximum auxin concentration at the tips of sepal and petal primordia directs organ initiation and outgrowth (Fig. 3A; Stages II–IV, marked with red gradient color) [44]. Moreover, the influence established by the initial dorsoventral auxin gradient may last and contribute to the final petal shape. Thus, the growth forces generated by the inflorescence axis and flower axis help to shape the petal together by means of auxin concentration gradients (Fig. 3B, marked with blue and red gradient colors, respectively). In late development stages, auxin signaling is localized to future vascular cells to regulate vasculature patterning [34, 44]. Multiple downstream pathways, including cell proliferation and differentiation and pigmentation, contribute to the final petal form by interacting with the IN factors. In the dorsal and lateral petals of a zygomorphic flower (as shown in Fig. 3), the two growth forces have non-overlapping patterns in a single organ, which results in this organ having different growth rates in different directions, and consequently organ internal asymmetry (Fig. 3B). Logically, not only the symmetry of petals, but also the symmetry in other floral organs, such as sepals or carpel locules, will be influenced in the same way [45, 46]. Although the logic of the patterning process seems to be increasingly clear, the current understanding of the molecular details of auxin in petal internal asymmetry remains unresolved. According to this model, the DV and IN pathways in zygomorphic flowers act synergistically to shape asymmetric petals. The future challenge, however, lies in bringing them into focus.

## Conclusion

Plant organs are generated from the shoot or floral meristems of flowering plants, and their temporal and spatial growth patterns are determined by their genetic makeup and identities. Organ asymmetry is an important developmental feature that is considered to have independently evolved many times during the evolution of flowering plants. However, in existing plants, asymmetrical organs are always arranged regularly in a symmetrically higher order and should have certain biological functions. In addition, it has been noted that asymmetric organs usually appear in pairs with two forms of handedness in many conditions. Thus, it can be presumed that the developmental mechanisms of organ internal asymmetry should be correlated with the molecular basis of handedness in plants.

Previous efforts to study mutants with altered petal shapes in the zygomorphic flowers of *A. majus*, *M. confertiflora*, and *P. sativum* have helped to identify some genetic factors controlling IN asymmetry. Nevertheless, unlike the DV pathway in zygomorphic flowers, which is determined by some key factors that have been verified in many lineages across angiosperms, there is no common molecular origin of the mechanisms regulating IN asymmetry. Moreover, the IN pathway is not linear, as these factors are not specific to IN regulation, but are integrated into other developmental paths, such as vascular patterning and cell proliferation.

Since antiquity, it has been known that flower organs, including petals, are all derived from leaves. Combined with the characteristics of the internal asymmetry in petals and leaves, it is easy to conclude that vascular patterning is a key hub in the network regulating the asymmetrical development of organs. Therefore, future research on IN asymmetry should focus on how these IN factors interact with the auxin signaling pathway to influence vascular patterning in these asymmetrical organs. In short, the mechanism for the establishment of organ internal asymmetry needs to be integrated into a stable system of knowledge that can be applied in any asymmetrical organ of any species.

## Acknowledgments

The authors thank Shuangwen Deng in South China Botanical Garden, Chinese Academy of Sciences for providing the photos of *Sida rhombifolia*, *Viola stewardiana*, *Saxifraga stolonifera,* and *Crinum moorei*. This work was supported by the National Science Foundation of China (31771345), Guangdong Basic and Applied Basic Research Foundation (2021A1515110137), and China Postdoctoral Science Foundation (2021 M701261).

## Author’s contributions

D.L. and X.L. conceived the ideas. Q.Y. compiled most of the information and led the writing, and all authors revised the manuscript extensively.

## Data availability

The authors declare that all of the data supporting the findings of this study are available within the paper.

## Conflict of interest

The authors declare that they have no conflicts of interest.
